# High-Performance Binocular Disparity Prediction Algorithm for Edge Computing

**DOI:** 10.3390/s24144563

**Published:** 2024-07-14

**Authors:** Yuxi Cheng, Yang Song, Yi Liu, Hui Zhang, Feng Liu

**Affiliations:** 1 Collaborative Innovation Center on Atmospheric Environment and Equipment Technology, Nanjing University of Information Science and Technology, Nanjing 210044, China; 202183370035@nuist.edu.cn (Y.C.); 202312490113@nuist.edu.cn (Y.S.); pm834725@student.reading.ac.uk (H.Z.); 2 Department of Computer Science, University of Reading, Whiteknights, Reading RG6 6DH, UK; 3 Institute of Semiconductors, Chinese Academy of Sciences, Beijing 100083, China; liufeng181@mails.ucas.ac.cn; 4 University of Chinese Academy of Sciences, Beijing 100089, China

**Keywords:** binocular disparity, edge computing, 3D convolution, activation function, practical

## Abstract

End-to-end disparity estimation algorithms based on cost volume deployed in edge-end neural network accelerators have the problem of structural adaptation and need to ensure accuracy under the condition of adaptation operator. Therefore, this paper proposes a novel disparity calculation algorithm that uses low-rank approximation to approximately replace 3D convolution and transposed 3D convolution, WReLU to reduce data compression caused by the activation function, and unimodal cost volume filtering and a confidence estimation network to regularize cost volume. It alleviates the problem of disparity-matching cost distribution being far away from the true distribution and greatly reduces the computational complexity and number of parameters of the algorithm while improving accuracy. Experimental results show that compared with a typical disparity estimation network, the absolute error of the proposed algorithm is reduced by 38.3%, the three-pixel error is reduced to 1.41%, and the number of parameters is reduced by 67.3%. The calculation accuracy is better than that of other algorithms, it is easier to deploy, and it has strong structural adaptability and better practicability.

## 1. Introduction

It is important to obtain the depth information of objects in many advanced vision tasks. Binocular stereo matching technology imitates human eyes, calculates the disparity and perceived depth by using the horizontal difference between the left and right cameras, then obtains three-dimensional scene information. It is a key technology and important topic in the field of stereo vision. Binocular stereo matching technology has been widely used in the fields of 3D ranging, 3D reconstruction [1], autonomous navigation [2], robot control [3], virtual reality [4], and so on. The binocular ranging method has wide application prospects because of its advantages of low cost, high precision, and simple deployment. Similarly to the development process of convolutional neural networks in other visual tasks, although the accuracy of stereo matching networks is improving, the depth of the networks is also increasing, followed by the rapid increase in the number of network parameters and increasing computational cost.

CASSANN-v2 [5], designed by the Institute of Semiconductors, Chinese Academy of Sciences, is a high-performance accelerator architecture with on-chip adaptive memory-tuning capabilities, enabling neural network acceleration for edge computing. Nowadays, with the rapid development of artificial intelligence, more and more intelligent applications need to be deployed on mobile and embedded devices, as well as edge computing devices. Existing deep learning-based disparity algorithms have made significant strides in the field of stereo matching. Jure and LeCun [6] designed a convolutional neural network that estimates disparity by calculating the similarity between image blocks. Fatma and Geiger [7] developed a network focused on resolving stereo ambiguities capable of predicting depths of textureless, reflective, and transparent surfaces. Pang et al. [8] proposed a two-stage convolutional network where the first stage uses an improved version of DispNet [9] for disparity estimation and the second stage corrects preliminary results. Eigen et al. [10] refined disparity map estimations by stacking two deep neural networks. Lena et al. [11] studied the mapping relationship between RGB images and depth maps through a supervised deep neural network, optimizing with Huber loss. Although capable of achieving high-precision disparity calculation, such methods typically suffers from high power consumption, high computational cost, and insufficient computational efficiency, making them unsuitable for deployment on edge computing devices like CASSANN-v2. Even lightweight networks contain modules that edge computing devices cannot directly deploy and, thus, need separate design considerations. Before being ported to edge computing devices, neural networks need to be optimized and improved to meet the requirements of being lightweight, efficient, and low-cost.

In order to solve the above problems, we propose a disparity calculation algorithm based on low-rank approximation and unimodal cost volume filtering. The algorithm proposed in this paper is optimized for the two aspects of computational complexity and network modeling ability, and a binocular disparity calculation algorithm suitable for high-performance terminals was designed to realize the trade-off between accuracy and calculation cost. The main contributions of this work are summarized as follows:

1. The three-dimensional convolution in the matching cost aggregation process is equivalent to one- and two-dimensional convolution according to the low-rank approximation principle, and the action mode of one- and two-dimensional convolution on the output is demonstrated and determined, which greatly reduces the number of network weights.
2. In terms of disparity accuracy, the activation function with pixel-level modeling capability is used to optimize the gradient propagation of the disparity computing network after network compression and address approximation to improve the performance of the network.In this way, for the edge computing device, only one convolution layer and one max operation are needed to achieve an activation function with pixel-wise modeling capability.
3. The matching cost volume is regularized by using unimodal cost volume filtering and a confidence estimation network, and the network parameters are updated by an independent loss function only in the training stage, which reduces the video memory in the running stage and alleviates the problem of the disparity matching cost distribution being far from the real distribution.

## 2. Related Works

### 2.1. Disparity Estimation

In recent decades, stereo matching technology has made significant progress, driven by the evolution from traditional algorithms to deep learning methods. Traditional stereo matching techniques typically involve the following four steps: matching cost computation, cost aggregation, disparity calculation, and disparity refinement [12]. Although effective, these methods are often limited by slow processing speeds and reduced accuracy, which restrict their wider application.

In recent years, the success of convolutional neural networks (CNNs) in various visual tasks, such as object detection and semantic segmentation [13], has encouraged researchers to apply deep learning to the field of stereo matching. For example, MC-CNN [14] was the first to apply CNNs to the computation of matching costs, calculating the similarity between stereo image pairs by extracting abstract features. This was followed by Mayer et al.’s introduction of DispNetC [9], an end-to-end stereo matching network that directly produces disparity maps from stereo images, marking a new direction in stereo matching research.

Additionally, GCNet (Geometry and Context Network) [15] introduces a novel approach by forming a cost volume from cascaded feature maps under different disparities, clearly representing the geometric features of the image. Then, 3D convolution is applied to this cost volume, extracting features across the dimensions of height, width, and disparity, which is crucial for learning environmental information and improving stereo matching results. PSMNet [16] expands on these ideas by integrating a deep residual network (ResNet) [17] for feature extraction and employing a Spatial Pyramid Pooling (SPP) structure [18]. This design enables the network to capture both global and local information at various scales, forming a comprehensive matching cost volume that significantly enhances the accuracy of disparity estimation.

The latest algorithms, like RAFT-Stereo [19] and HitNet [20], continue to advance the field. RAFT-Stereo utilizes a recursive multi-scale approach, progressively refining the disparity map across different iterations, enhancing both accuracy and convergence speed. Meanwhile, HitNet, through its hierarchical iterative tile network, focuses on efficiently resolving disparity issues within small regional blocks, achieving high precision and efficiency in complex scenes.

These developments demonstrate that the integration of deep learning with stereo matching algorithms not only significantly improves speed and accuracy but also expands their potential applications in areas such as autonomous driving, robotic navigation, and augmented reality. With ongoing technological advancements and the introduction of new datasets, the future of stereo matching technology looks increasingly widespread and efficient.

### 2.2. Three-Dimensional Convolution and Its Optimization

The application of 3D convolution for the extraction of spatio-temporal features from video data was originally proposed by Ji et al. [21]. This pioneering approach laid the groundwork for subsequent innovations in 3D convolutional neural networks (CNNs), such as the C3D model developed by Tran et al. [22] for human action recognition. This model has since become a benchmark within the field, demonstrating the significant advantages of 3D convolutional techniques over traditional 2D convolution in analyzing complex video data.

Building on this foundational work, Hara et al. [23] further advanced the application of 3D convolution by integrating it into the ResNet101 [17] architecture. This integration significantly enhanced the model’s capabilities, allowing for more detailed and precise temporal and spatial analyses of video sequences. Such advancements underscore the superiority of 3D convolution in capturing the dynamic and intricate patterns of movement and behavior within video data.

Despite the efficacy of 3D convolution in handling video data, its adoption in real-world applications has been limited by the high computational cost associated with its algorithmic complexity. This complexity introduces a significant overhead, making it challenging to deploy these models in environments where processing efficiency is paramount [24].

In response to these computational challenges, the research community has explored various strategies to mitigate the intensive demands of 3D convolution. One notable approach has been the development of hybrid models that amalgamate the spatial analytical power of 2D convolutions with the temporal depth of 1D convolutions. These hybrid models aim to optimize processing requirements while maintaining robust feature extraction capabilities, thus addressing the dual needs of efficiency and effectiveness.

Among the innovative solutions in this area, the Pseudo-3D Residual Network (P3D ResNet) developed by Qiu et al. [25] represents a significant breakthrough. This architecture cleverly decomposes traditional 3D convolutions into a combination of 2D and 1D operations, thereby reducing both computational load and parameter count. While the P3D model has successfully reduced computational demands, its impact on processing speeds has been constrained by limited compression efficiency.

The ongoing research and development in 3D convolutional technologies reflect a concerted effort to strike an optimal balance between the depth of feature extraction and computational efficiency. This balance is crucial for the broader adoption of these technologies in real-time applications such as video surveillance, interactive media, and sports analytics. With continued advancements, 3D convolutional networks are expected to become even more effective and feasible for widespread use, paving the way for smarter and more responsive video analysis systems.

## 3. Methods

**Figure 1:**
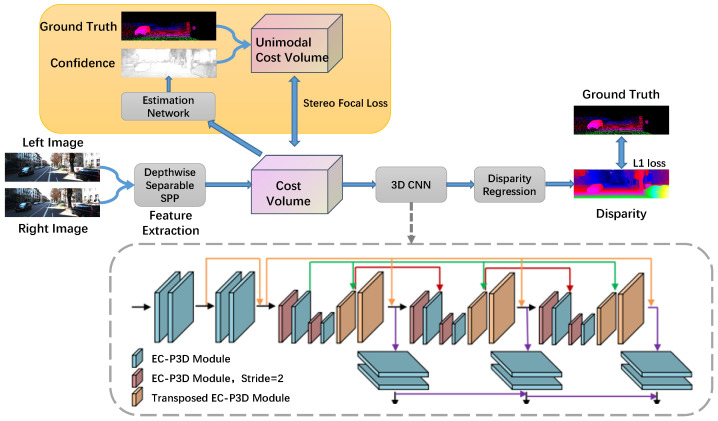
Framework of the proposed algorithm.

Binocular disparity calculation algorithm design usually includes the following four steps: feature extraction, matching cost calculation and aggregation, disparity calculation, and disparity refinement. In contrast to traditional methods, each module needs to be independently designed and trained. The algorithm proposed in this study is an end-to-end binocular disparity calculation algorithm based on a deep neural network, and the gradient can be transmitted between each module. The weight of the network is updated by the supervised training loss function. The framework of the algorithm proposed in this study is shown in Figure 1. The entire process is conducted through a backbone network, as shown in Figure 1 by the unboxed middle line (outside of the yellow background box above and the dashed gray line box below). Below, we first introduce the overall structure, then detail the advantages of our algorithm.

### 3.1. Overview

Our network’s process involves inputting left and right stereo images into a CNN network that shares weights and is used to compute the feature map. These feature maps are then fed into a depth-separable SPP (spatial pyramid pooling) module for feature extraction. The first part of the SPP module processes the input images in sub-regions of varying sizes, and the results are then concatenated with the output of a convolution layer designed for feature fusion, producing merged features from both the left and right images. Subsequently, these features are used to construct a four-dimensional matching cost volume. This cost volume is then processed by a pseudo 3D module designed for edge computing (EC-P3D) and a transposed EC-P3D module to complete cost aggregation and disparity regression. During training, the loss between the network output and the actual labels (L1) is continuously calculated to optimize the network parameters, ultimately yielding the predicted disparity.

### 3.2. Pseudo 3D Convolution

The four-dimensional feature vector of [C,D,H,W] can be obtained after matching cost calculation. In order to fuse the context relationship between the spatial domain and the disparity domain, 3D convolution with a nonlinear activation function (ReLU) and batch normalization is needed to learn the features defined in this dimension. In contrast to 2D convolution used for 3D feature vectors of [C,H,W], the number of parameters and computational complexity of 3D convolution used for 4D feature vectors of [C,D,H,W] are significantly increased, taking up a lot of computational resources and making training more difficult. Therefore, in order to reduce the number of parameters and computational complexity of 3D convolution, this study uses matching cost aggregation based on low-rank approximation for 3D convolution. Table 1 shows a comparison of the number of parameters and the computational complexity after 3D convolution low-rank approximation.

For a video clip, we can abstract it as a tensor of size C×L×H×W, where C,L,H, and *W* represent the channel number, frame number, frame height, and frame width, respectively. The most direct way to extract the tensor is using 3D convolution, which can model spatial information and extract time sequence information between frames. Suppose we have a 3×3×3 convolution kernel, which can be naturally decomposed into a 2D convolution kernel of 1×3×3 in the space domain and a 1D convolution kernel of 3×1×1 in the time domain. In this way, the decomposition of 3D convolution can greatly reduce the size of the model.

Based on the above ideas, P3D block extends the 2D residual unit in ResNet and realizes the spatio-temporal coding of video in a structure similar to that of ResNet. ResNet’s residual network consists of a large number of residual units, which can usually be expressed in the following way:

(1) $$\begin{array}{c} x_{t+1}=h\left(x_{t}\right)+F\left(x_{t}\right) \end{array}$$

where $x_{t}$ and $x_{t+1}$ represent the input and output of the residual element, respectively; $h\left(x_{t}\right)=x_{t}$ represents the identity mapping; and F is a nonlinear residual function. ResNet, with a shortcut, no longer learns a nonlinear function directly from input to output but from the residual between the output and the input.

**Figure 2:**
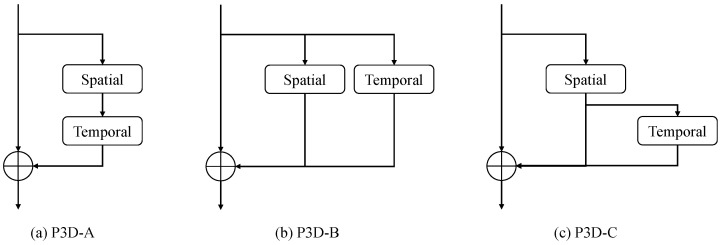
Three designs of P3D blocks.

The design idea of P3D block is to expand all the convolution kernels in the above 2D residual unit into 3D, then decompose the 3D convolution kernels into 1×3×3 a 2D convolution and a 3×1×1 1D time convolution. Since the original 3D convolution was decomposed into two filters, it is necessary to consider whether the result of the spatial convolution is directly input to the time convolution or whether it is carried out in parallel and not in sequential order. Another point that needs to be considered is whether the results of these two convolution kernels directly affect the output results of the residual element. Based on the above two design ideas, P3D designs three block structures [25], namely P3D-A, P3D-B, and P3D-C. A schematic of the three structures is shown in Figure 2.

P3D-A: As shown in Figure 2a, P3D-A first performs a 2D spatial convolution followed by a 1D temporal convolution. These two convolutions are directly connected, with only the 1D temporal convolution and the final output connected. The relationship between input $x_{t}$ and output $x_{t+1}$ can be represented as follows:

(2) $$\begin{array}{c} \left(I+T\cdot S\right)\cdot x_{t}:=x_{t}+T\left(S\left(x_{t}\right)\right)=x_{t+1} \end{array}$$

where *I* indicates an identity mapping.

P3D-B: As shown in Figure 2b, P3D-B performs 2D spatial convolution and 1D temporal convolution simultaneously, the results of which are added together. The relationship between input $x_{t}$ and output $x_{t+1}$ can be represented as follows:

(3) $$\begin{array}{c} \left(I+S+T\right)\cdot x_{t}:=x_{t}+S\left(x_{t}\right)+T\left(x_{t}\right)=x_{t+1} \end{array}$$

P3D-C: As shown in Figure 2c, P3D-C is a compromise between P3D-A and P3D-B. It first performs a 2D spatial convolution, followed by a shortcut branch that combines the results of the 2D spatial convolution and 1D temporal convolution. The relationship between input $x_{t}$ and output $x_{t+1}$ can be represented as follows:

(4) $$\begin{array}{c} \left(I+S+T\cdot S\right)\cdot x_{t}:=x_{t}+S\left(x_{t}\right)+T\left(S\left(x_{t}\right)\right)=x_{t+1} \end{array}$$

In the process of stereo disparity calculation, the spatial features from the left and right images need to be matched to produce disparity outputs. The results are not directly influenced by the three different structures, but based on experimental evidence, structures 1 and 3 are capable of performing this task. However, structure 3 involves too many connections, making it unsuitable for data transmission on chips. Therefore, structure 1 is adopted. This choice resolves issues related to the chip’s inability to support 3D convolutions and the problem of excessive parameter volume, aligning with the characteristics of disparity calculation algorithms.

### 3.3. WReLU Activation Function with Pixel-Level Modeling Capability

**Figure 3:**
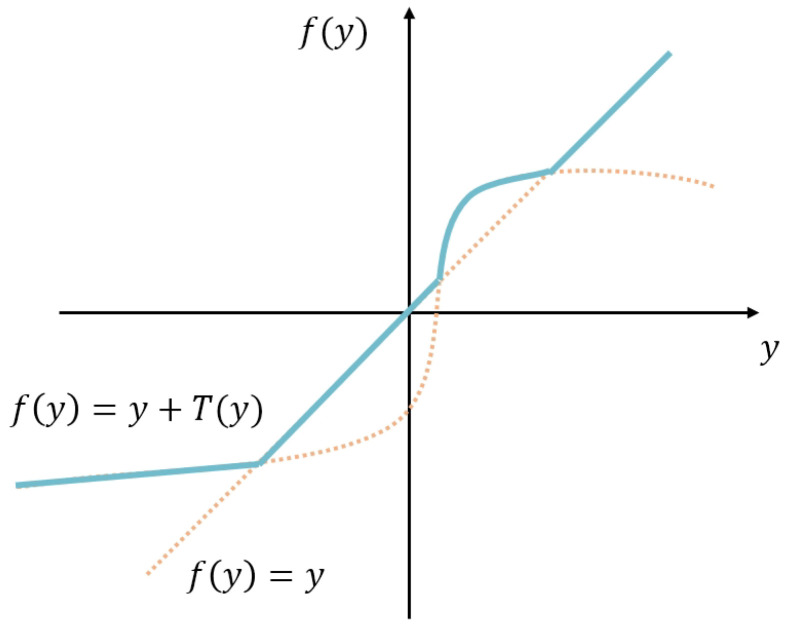
An example of the WReLU activation function (the blue line for a possible case, the orange dashed line is the function interval that is ignored after WReLU is formed).

In this study, the WReLU activation function [26] is used to avoid the problem of information loss and to ensure pixel-level modeling capability. WReLU extends the ability of the ReLU activation function by adding a residual space condition at a negligible additional computational cost. The algorithm can generate complex gradients and achieve a better training process without the gradient disappearing, so it has an advantage in terms of performance. For each input pixel ($x_{i,j}$), WReLU sets an operation window centered at $x_{i,j}$. The size of the window is *k* × *k*, and our calculation first adopts a inner vector product where pixels in the window are viewed as a vector ($p_{i,j}$).

(5) $$\begin{array}{c} T\left(x_{i,j}\right)=BN\left(u\cdot p_{i,j}\right) \end{array}$$

where *u*, containing k×k learning parameters elements in windows, is a channel-wise parameter to reduce the number of network parameters and *BN* represents the batch normalization operation. Then, WReLU is defined as follows:

(6) $$\begin{array}{c} W\left(x_{i,j}\right)=\max\left(x_{i,j},x_{i,j}+T\left(x_{i,j}\right)\right) \end{array}$$

where $T\left(x_{i,j}\right)$ represents the residual difference of the function fitted by *x* and the network, and max(·) makes the activation function nonlinear and ensures pixel-level modeling capability. A schematic and the expression of our activation function are shown in Figure 3.

WReLU improves the generalization performance of the model by adding parameters. It introduces windows to capture spatial dependencies for better presentation. This algorithm can generate complex gradients and achieve a better training process without gradient disappearance, so it has advantages in terms of performance.

### 3.4. EC-P3D Module and Transposed EC-P3D Module

A lightweight stereo matching module, EC-P3D, based on pseudo 3D convolution and low-rank approximation, is proposed for high-performance terminals. This module enables the deployment of disparity calculation algorithms on AI chips that cannot directly implement 3D convolution. Replacing the bulky, computationally intensive, and hard-to-deploy 3D and transposed 3D convolutions with this new module reduces the number of network parameters while maintaining network accuracy.

**Figure 4:**
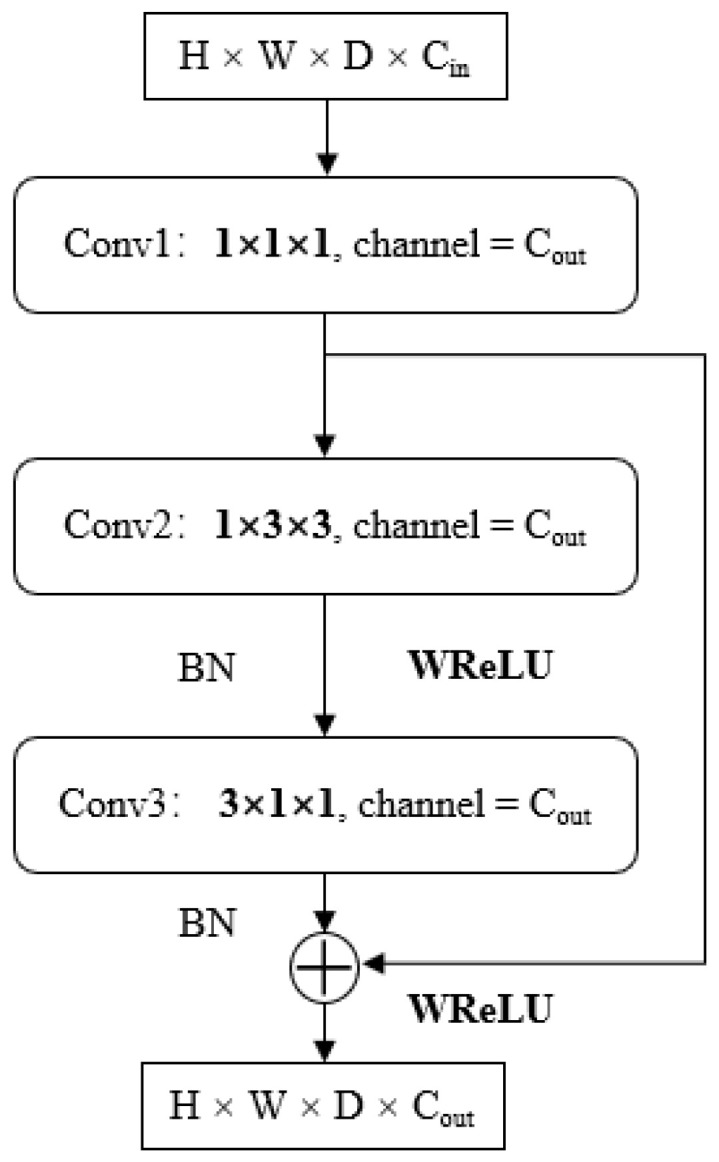
EC-P3D module.

EC-P3D consists of two main parts, as shown in Figure 4. The design of the first part is inspired by P3D and is based on low-rank approximation, using 1D and 2D convolutions to approximate the 3D convolution. However, there are significant differences between them. Unlike P3D-A, we use Conv1 for channel compression of the input feature map to avoid taking up too much video memory. Then, the compressed feature map is combined with the output of the subsequent pseudo 3D module to obtain the final result. In the pseudo 3D part, we first use 3 × 3 convolution to perform 2D convolution on each compressed feature map of size H × W in dimension D, then stack the results of each dimension on dimension D to generate new feature map and change the data index method of the feature map. A 3 × 1 × 1 convolution kernel (size 3 in dimension D) is used to conduct 1D convolution for the newly generated feature map; then, the generated 1D result is resized as H × W so as to achieve the equivalent of 3D convolution.

Due to the equivalency of the process, the new convolution kernel loses a large amount of correlation information inside the 3D tensor during feature extraction (that is, the correlation of pixels at different corresponding positions in each feature map in dimension D within the 3 × 3 × 3 element area), so we use WReLU to restore this correlation pixel by pixel. Meanwhile, in order to further reduce the number of parameters in the model and improve the realizability of chip deployment, basic 2D blocks of feature extraction are replaced with depth-wise convolution similar to MobileNet.

**Figure 5:**
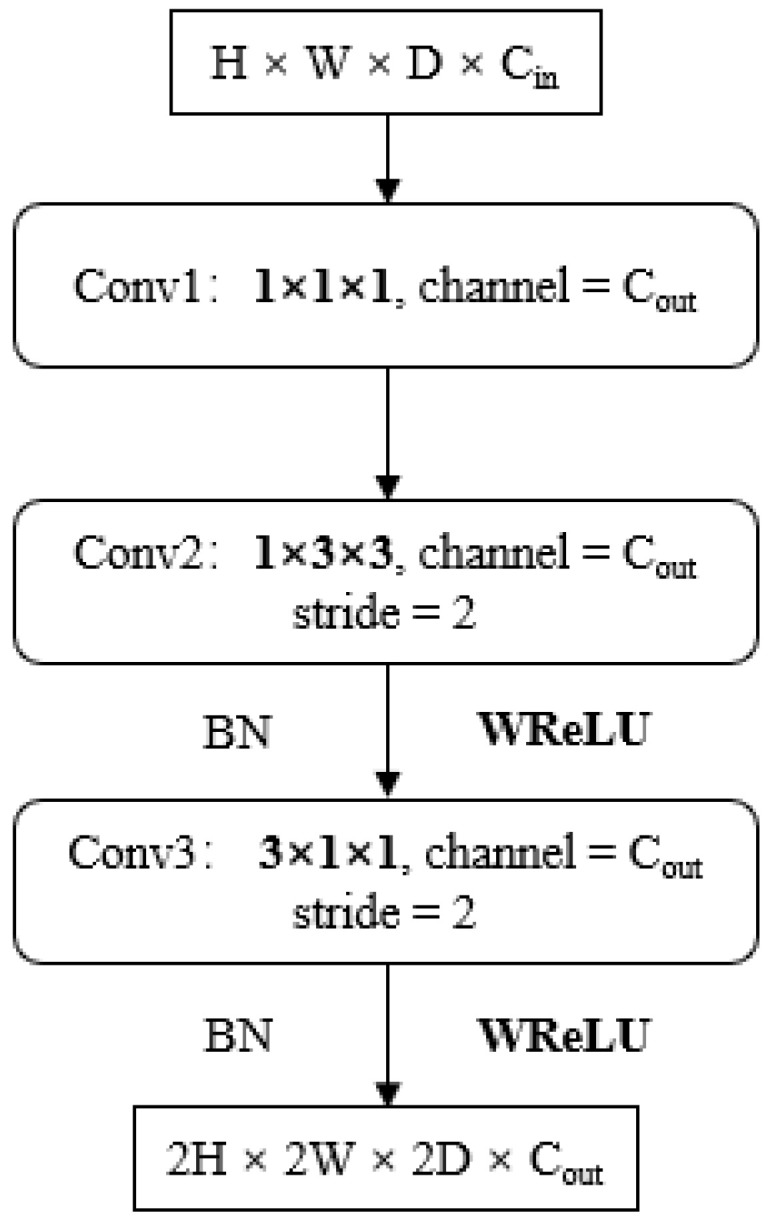
Transposed EC-P3D module.

As up-sampling cannot be completed by a CNN chip directly and FPGA is needed for auxiliary design, in order to realize up-sampling of the feature map, we propose a transposed EC-P3D module. The transposed EC-P3D module is shown in Figure 5. Since transposed 3D convolution actually implements the up-sampling process, the transposed EC-P3D module does not require a shortcut. Like the EC-P3D module, Conv2 and Conv3 are composed of a 1 × 3 × 3 2D convolution kernel and a 3 × 1 × 1 1D convolution kernel, applying dimension raising in the H, W, and D dimensions, respectively. The final results are obtained through *BN* and WReLU.

For the data storage of edge computing devices, 1 × 3 × 3 and 3 × 1 × 1 are equivalent to the 3 × 3 2D convolution and the 1D convolution of size 3, which greatly reduces the difficulty of deployment and improves the utilization of the chip.

### 3.5. Adaptive Unimodal Cost Volume Filtering

**Figure 6:**
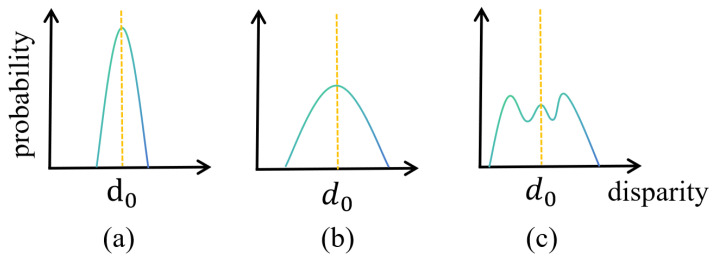
Three types of matching probability distribution, where the blue is the disparity probability distribution curve, and the orange dashed line is the predicted disparity value.

Matching cost volumes indirectly supervised by disparity regression tend to be highly ambiguous; an infinite number of matching cost distributions can produce the same disparity regression results. Specifically, the three types of matching probability distributions illustrated in Figure 6a–c can all yield the same disparity values. However, only distributions (a) and (b)—where the peak is sharp and high at the true disparity, indicating low uncertainty—are considered reasonable.

We demonstrate a typical pixel’s matching cost distribution, where a bimodal distribution also yields the correct disparity. This flexibility in matching cost, which lacks direct supervision, means that incorrectly learned cost volumes can still approximate true disparities, leading to severe overfitting and reduced network accuracy. Therefore, it is necessary to regularize the matching cost based on its unimodal characteristics to align the cost distribution more closely with the true distribution. We propose unimodal cost volume filtering and a confidence estimation network for to regularize the matching cost volume.

In this study, we filter the matching cost volume with the unimodal distribution that peaks at the real disparity and add constraints directly to the matching cost by adding a regularized network. In addition, the network estimates the variance of the unimodal distribution of each pixel and explicitly models the uncertainty of matching in different environments. In order to avoid the problem of the video memory being too large to run during network inference, we designed it as a plug-and-play module that only needs to supervise the matching cost during training and can shield this part of the network during inference and only use the subject network weight optimized by this module so as to reduce the video memory required during the inference stage.

Given the true disparity ($d^{gt}$), the unimodal distribution is defined as follows:

(7) $$\begin{array}{cc} P(d) & =\mathrm{Softmax}\left(-\frac{\left|d-d^{gt}\right|}{\sigma }\right)=\frac{\exp\left(-c_{d}^{gt}\right)}{\sum_{d^{\prime }=0}^{D-1}\exp\left(-c_{d^{\prime }}^{gt}\right)} \end{array}$$

(8) $$\begin{array}{c} c_{d}^{gt}=\frac{\left|d-d^{gt}\right|}{\sigma } \end{array}$$

where σ>0 is the variance, which controls the sharpness of the peaks around the true disparity.

However, the matching cost volume constructed by P(d) based on the above method and serving as a true label has the same peak sharpness (i.e., variance) between different pixels, which cannot reflect the differences in the distributions to which different pixels belong. In order to construct a more reasonable real label for the matching cost volume, a confidence estimation network ($f_{p}$) is added to the algorithm to adaptively predict the variance ($\sigma_{p}$) of the corresponding distribution of each pixel.

(9) $$\begin{array}{c} \sigma_{p}=s\left(1-f_{p}\right)+\epsilon \end{array}$$

where s≥0 is the scale factor reflecting the sensitivity of $\sigma_{p}$ to the change in confidence ($f_{p}$) and ϵ>0 defines the lower bound of $\sigma_{p}$, avoiding the numerical problem of division by 0. Thus, $\sigma_{p}\in \left[\epsilon ,s+\epsilon \right]$. Untextured pixels and blocked pixels have larger $\sigma_{p}$ values because untextured pixels tend to have multiple matches, while blocked pixels do not have the right match. Pixel-by-pixel adaptive estimation ($\sigma_{p}$) can modify the cost volume of the real tag defined in the Formula (7) to generate a standard cost volume as a supervised training tag, namely

(10) $$\begin{array}{cc} P(d) & =\mathrm{Softmax}\left(-\frac{\left|d-d^{gt}\right|}{\sigma_{p}}\right). \end{array}$$

Based on the above discussion, for pixel position *p* in the matching cost volume, there is a matching cost distribution (${\hat{P}}_{p}\left(d\right)$) produced by the network estimation and the real label ($P_{p}\left(d\right)$). The loss function between them can be defined by cross-entropy, and the serious sample imbalance problem needs to be solved [6]. Stereo focal loss is used in binocular disparity calculation to focus the loss function on the positive disparity sample to avoid the total loss dominated by the negative disparity sample.

(11) $$\begin{array}{c} L_{SF}=\frac{1}{\left|P\right|}\sum_{p\in P}\left(\sum_{d=0}^{D-1}{\left(1-P_{p}\left(d\right)\right)}^{-\alpha }\cdot \left(-P_{p}\left(d\right)\cdot \log{\hat{P}}_{p}\left(d\right)\right)\right) \end{array}$$

where α≥0 is a parameter that represents the degree of focus. When α=0, the stereo matching focus loss degenerates into cross-entropy loss, and when α>0, more weight can be proportionally assigned to the positive disparity sample by $P_{p}\left(d\right)$. Therefore, simple negative disparity samples are further explicitly suppressed at considerably smaller weights, and positive disparity samples are allowed to compete with only a few difficult samples.

### 3.6. Loss Function of Multi-Module Fusion Training

The disparity-computing network discussed in this chapter consists of two loss functions. The first regression loss function is defined between the predicted disparity map and the true disparity map and divided into three stages, as shown in the structure within the gray dotted box in Figure 1. The output disparity map of all stages is supervised, supervising the entire network. The second stereo matching focus loss function is defined in a supervised way between the generated real matching cost and the matching cost obtained by network matching.

For the loss of the predicted disparity map and the real disparity map, the $smoothL_{1}$ loss function in the first *k* stages is defined according to the real disparity ($d_{p}$) of each pixel *p*, namely

(12) $$\begin{array}{c} L_{k}=\frac{1}{\left|P\right|}\sum_{p\in P}smoothL_{1}\left(d_{p}-{\hat{d}}_{p}\right) \end{array}$$

(13) $$\begin{array}{c} smoothL_{1}\left(x\right)=\left\{\begin{array}{cc} 0.5x^{2}, & \;\mathrm{if}\;|x|<1\\ |x|-0.5, & \;\mathrm{otherwise} \end{array}\right., \end{array}$$

where the predicted disparity value is ${\hat{d}}_{p}$, and P represents the set of points with true disparity in the label. The $smoothL_{1}$ loss function is insensitive to outliers and can remain robust at outliers in the disparity graph (e.g., noise, etc.). For each phase, the $smoothL_{1}$ loss function is used to measure the error between the predicted disparity map and the true disparity map. The entire regression loss function is the weighted sum of the losses at each stage, namely

(14) $$\begin{array}{c} L_{\mathrm{reg}}=\sum_{k=1}^{3}\lambda_{k}\times L_{k}, \end{array}$$

where $\lambda_{k}$ is the weight of regression loss in stage *k*, and the weight values of the three stages are 0.25, 0.5, and 1.0, respectively.

For stereo matching focus loss, in order to make more pixels tend toward high confidence values, it is necessary to add $L_{\mathrm{conf}}$ as the regularization term, as follows:

(15) $$\begin{array}{c} L_{\mathrm{conf}}=\frac{1}{\left|P\right|}\sum_{p\in P}-\log f_{p} \end{array}$$

In summary, the loss function of the binocular disparity calculation algorithm based on low-rank approximation and unimodal cost volume filtering is defined as follows:

(16) $$L_{all}=\lambda_{\mathrm{reg}}L_{\mathrm{reg}}+L_{SF}+\lambda_{\mathrm{conf}}L_{\mathrm{conf}},$$

where $\lambda_{\mathrm{reg}}$ and $\lambda_{\mathrm{conf}}$ are hyperparameters used to balance the weight of various loss functions. $L_{\mathrm{reg}}$ supervises disparity training, and $L_{SF}$ supervises cost volume.

## 4. Experiments

In order to verify the performance of the disparity calculation algorithm based on low-rank approximation and single-peak cost body filtering proposed in this chapter, relevant comparative experiments were designed on public datasets. First, information on two related datasets (SceneFlow [9] and KITTI 2015 [27]) is presented. Secondly, the implementation details and training strategies of the network are introduced in detail. Finally, different network structures are ablated to test the influence of network structure and parameter settings on the results.

### 4.1. Datasets

The datasets used in this chapter are the large SceneFlow and KITTI 2015 datasets containing pictures and real disparity values, 80% of which are randomly selected as the training set and the remaining 20% as the test set. SceneFlow is a synthetic dataset. The training set contains 168,357 stereo images, and the test set contains 19,854 stereo images to test model performance in the training stage. KITTI 2015 is a dataset collected from the real world with a maximum disparity of 192, a training set containing 400 images, and a validation set containing 800 images without real labels. This chapter uses SceneFlow for pre-training and testing, with the model trained, fine-tuned, and tested on KITTI 2015.

### 4.2. Training Details

NVIDIA TitanXp GPUs with 11G video memory in the Ubuntu environment using the PyTorch deep learning framework are used to implement the EC-P3D module proposed in this study. The model is trained end-to-end using 4 NVIDIA TitanXp GPUs with 11G video memory, the batch size set to 4 and the Adam optimizer. For all datasets, the size of training images is set as 512 × 256, the RGB values of all images are normalized to the range of [−1, 1], and the maximum disparity value ($D_{max}$) is set as 192. For the SceneFlow dataset, 10 epochs are trained at a fixed learning rate of 0.001. For the KITTI 2015 dataset, the model pre-trained on the SceneFlow dataset is used in this study for further optimization training. There are 300 epochs of optimization training, among which the learning rate is 0.001 in the first 200 epochs and adjusted to 0.0001 in the last 100 epochs.

### 4.3. Metrics

Since we use multiple depth estimation datasets and there are various metrics for evaluating different datasets we list the metrics we use.

End-point error (EPE): End-point error is used in the evaluation of the SceneFlow dataset. Formally, the difference between the result and the ground truth can be written as $EPE\left(d^{*}-\hat{d}\right)={∥d^{*}-\hat{d}∥}^{2}$, where $d^{*}$ is the result of the network output, and $\hat{d}$ is the ground truth.

The percentage of erroneous pixels: The percentage of error pixels is used in the evaluation of the KITTI 2012 and KITTI 2015 datasets. Specifically, a pixel is considered an error pixel when its disparity error is greater than *t* pixels. Then, the percentage of error pixels in the non-occluded area (Out-NOC) and the total area (Out-all) is calculated. Specifically, for KITTI 2012, t∈{2,3,4,5}. For KITTI 2015, a pixel is considered wrong when the disparity error is greater than three pixels or 5%.

### 4.4. Results and Evaluation

In the comparison experiment, first, the performance of this algorithm is compared quantitatively and qualitatively with other dense disparity-computing algorithms; then, three improvements are proposed for this research. Ablation experiments on the WReLU activation function, EC-P3D adaptation module, and matching cost regularization are conducted to test the influence of network structure and parameter settings on the results. Finally, the video memory occupancy of the network is tested.

#### 4.4.1. Qualitative Analysis and Comparison of Algorithm Performance

**Figure 7:**
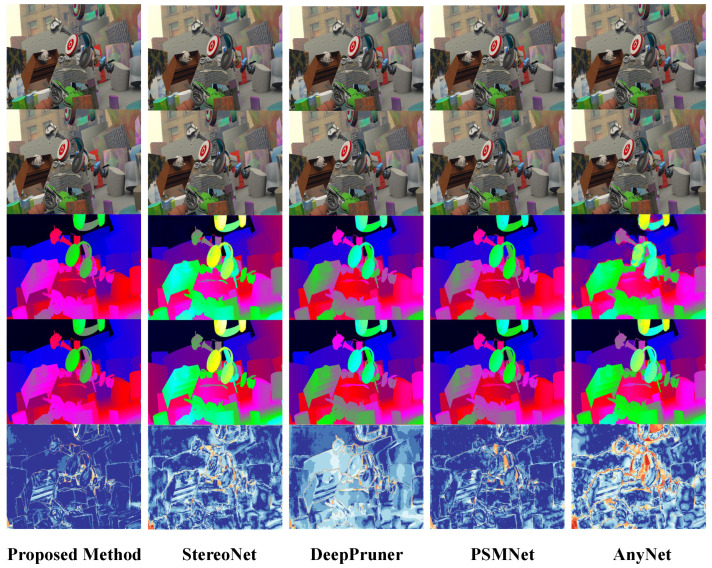
Visualization results of the algorithm proposed in this chapter on the SceneFlow dataset.

For pre-training on the SceneFLow dataset, Figure 7 shows a comparison between the proposed algorithm and other algorithms based on deep learning, where the first and second lines represent the input left and right stereo image pairs, the third line represents the network prediction results, the fourth line represents the true disparity, and the last line is the error heat map. The colder the heat map, the lower the error, and the warmer the color, the higher the error. The use of the heat map can make the error more clearly visible. Therefore, the color of the occluded area in the error heat map is warmer.

**Figure 8:**
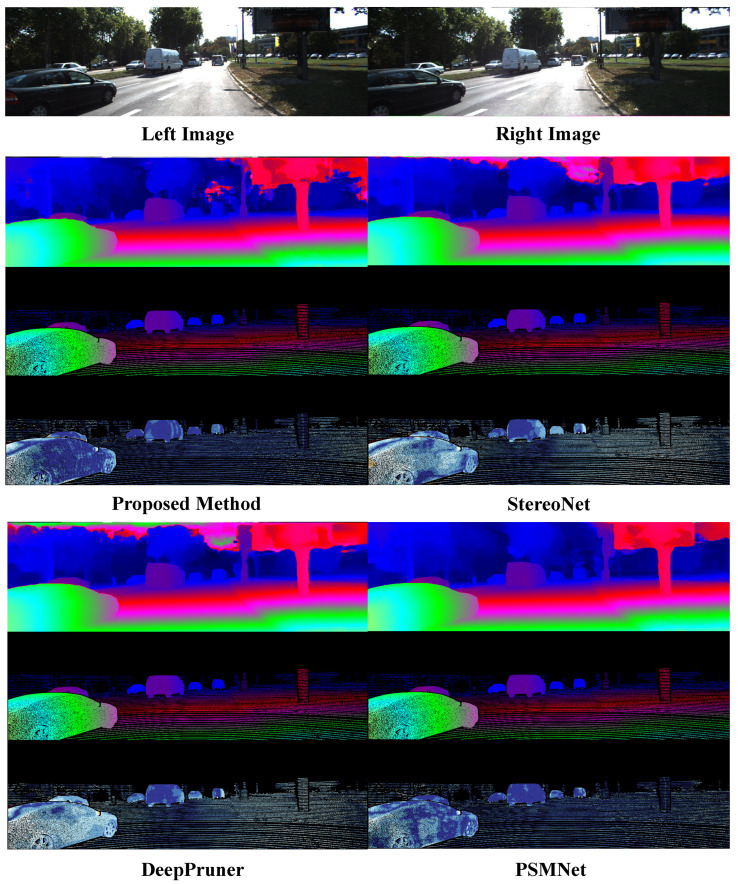
Visualization results of the algorithm proposed in this chapter on the KITTI 2015 dataset.

**Figure 9:**
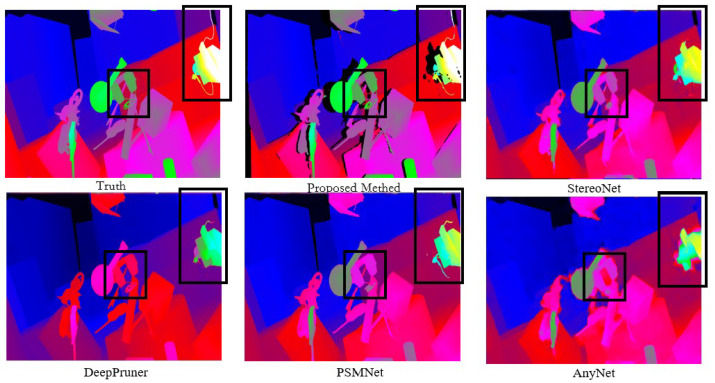
The ability to represent fine structures and edges.

**Figure 10:**
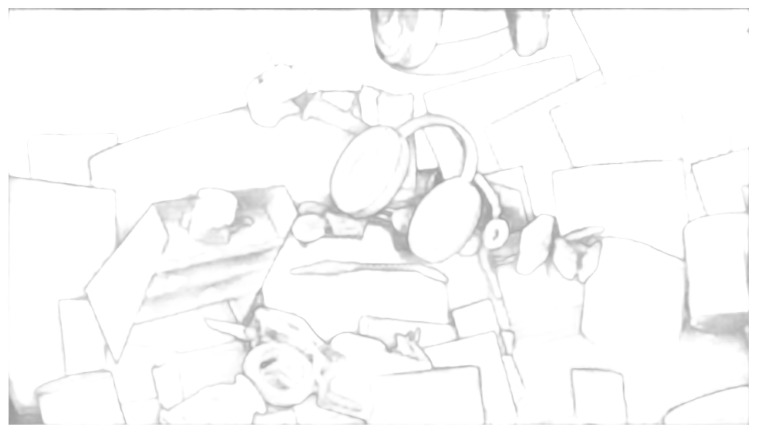
Confidence network output on the SceneFLow dataset.

**Figure 11:**
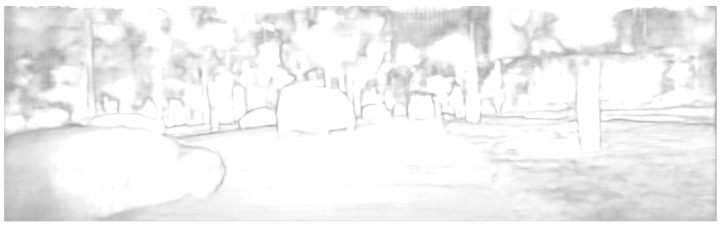
Confidence network output on the KITTI 2015 dataset.

For fine tuning on the KITTI dataset, Figure 8 shows a comparison of the algorithm proposed in this study with other deep learning-based algorithms in real-world scenarios. According to the analysis of the experimental results presented in Figure 7 and Figure 8, the following conclusions can be drawn:

(1) In the error heat map, the overall color of the algorithm proposed in this study is more cool, so the overall accuracy is higher than that of other compared algorithms.
(2) The output of the disparity map is dense, and the disparity changes continuously in the semantically relevant regions. Although the algorithm’s adaptation of compression and low-rank approximation is performed on the terminals, the quality of the disparity output is still guaranteed.
(3) Due to the regularization operation on the matching cost volume, the algorithm proposed in this study achieves sharper and clearer boundaries in the edge areas compared to other algorithms. As shown in the black box in Figure 9, our algorithm demonstrates superior capabilities in representing fine structures and edges over other networks. The output of the confidence network is shown in Figure 10 and Figure 11, which not only represent the reliability of the disparity predicted by the network for these pixels but also objectively reflect the possibility of the real scene point corresponding to the pixel being in the edge, occlusion, or subtle structure.

#### 4.4.2. Quantitative Analysis and Comparison of Algorithm Performance

Table 2 shows the results of quantitative analysis of the absolute error index and three-pixel error index of the algorithm proposed in this study on SceneFlow dataset and the KITTI dataset, as well as the quantitative analysis results of other algorithms under the same evaluation criteria. When testing the algorithm, the average value obtained by repeating 10 experiments is used to evaluate the index. The test of running time is based on the experimental platform proposed in this study, but this index is directly related to the performance of the CPU, so it is only used as a comparison of running time between algorithms and is only for reference for other operating platforms.

By analyzing the data in Table 2, it can be seen that the algorithm proposed in this study is significantly superior to other methods in terms of the end-point error index (EPE) and three-pixel error and has greater advantages in terms of the number of parameters and running time compared with high-precision disparity calculation networks such as PSMNet and GC-Net. Although the running time on the current platform presents no obvious advantage compared with the lightweight algorithm, acceleration through the new transformer terminal offers the the possibility of real-time operation in the future.

#### 4.4.3. Ablation Study

In order to test the contributions of the proposed sub-modules of network compression, WReLU, and cost volume regularization to the overall binocular disparity calculation algorithm, ablation experiments were designed. For PSMNet and AnyNet, two classical high-precision and lightweight disparity calculation networks, starting from the basic structure, were added one by one according to the proposed order, and the network with different modules added was evaluated. The experimental results are shown in Table 3 and Table 4. Both network compression and EC-P3D modules significantly reduce the number of parameters and running time of the network and play the role of a lightweight network and hardware adaptation with less loss of accuracy. WReLU and cost volume regularization greatly improve the performance of the network, with considerable accuracy improvements, while the computational cost is almost unchanged, meeting the application requirements of high-performance terminals.

We conducted a performance analysis of the WReLU activation function on foundational vision models including SqueezeNet 1.0, SqueezeNet 1.1, SqNxt-23, MobileNetV2, and ShuffleNetV2 × 0.5. Research on and improvements of these models can be extended to other visual tasks, assisting in achieving better network accuracy for various applications. Therefore, this section conducts tests on these foundational models to verify the versatility of the WReLU activation function. The tests conducted across these five different visual classification base models all achieve excellent performance, as shown in Table 5. The WReLU activation function significantly enhances the network’s top-two and top-five accuracy rates, with almost no change in the number of parameters.

## 5. Conclusions

For the application scenarios of high-performance edge computing devices with high computing power, this paper conducts research on algorithm performance optimization based on hardware resource adaptation and proposes a disparity calculation algorithm based on low-rank approximation and unimodal cost volume filtering. In the matching cost aggregation part, an EC-P3D network structure is proposed. The three-dimensional convolution is equivalent to two-dimensional and one-dimensional convolution using low-rank approximation technology, which greatly reduces the number of network weights; in terms of disparity accuracy, a WReLU activation function with pixel-level modeling ability is proposed, which avoids information disappearance during backpropagation learning and enhances the expression ability of the network. This activation function only requires one layer of convolution and one size comparison for mobile chips; it is proposed to use unimodal cost volume filtering and confidence estimation network to regularize the matching cost volume, which alleviates the problem of the disparity matching cost distribution being far away from the true distribution. The unimodal cost volume filtering and confidence estimation network is a plug-and-play module that can be combined with other recent methods based on cost volume to continuously enhance the performance of depth estimation. Future developments are expected to focus on hardware adaptation and algorithm optimization based on transformers for depth estimation algorithms, which will open up broader application prospects.

Compared with the typical deep learning disparity calculation network, PSMNet, our proposed disparity calculation algorithm based on low-rank approximation and unimodal cost volume filtering reduces absolute error by 38.3%, three-pixel error to 1.41%, and the number of parameters by 67.3%. The calculation accuracy is better than that of other algorithms, and it is easier to deploy. The number of network parameters and computational cost are greatly reduced, and it has better practicability.

## Figures and Tables

**Table 1:** Comparison of 3D convolution with low-rank approximation.

Name	Parameters	Computational Complexity
Standard 3D convolution	$D^{\prime }DF_{T}F_{H}F_{W}$	$O\left(D^{\prime }DTHWF_{T}F_{H}F_{W}\right)$
2D convolution in the spatial direction	$D^{\prime }DF_{H}F_{W}$	$O\left(D^{\prime }DTHWF_{H}F_{W}\right)$
1D convolution in the temporal direction	$D^{\prime }DF_{T}$	$O\left(D^{\prime }DTHWF_{T}\right)$
Spatial + temporal	$D^{\prime }D\left(F_{H}F_{W}+F_{T}\right)$	$O\left(D^{\prime }DTHW\left(F_{H}F_{W}+F_{T}\right)\right)$

**Table 2:** Algorithm performance comparison.

	EPE	Three-Pixel Error%	Parameters	Running Time
PSMNet	1.09	4.35	5.2 M	0.50 s
AnyNet	3.19	6.20	0.04 M	97.3 ms
DeepPruner	0.86	2.15	N/A	182 ms
AANet	0.87	2.55	N/A	62 ms
AcfNet	0.86	1.89	5.6 M	0.48 s
GC-Net	2.51	2.87	3.5 M	0.95 s
Proposed method	0.77	1.41	1.7 M	0.48 s

**Table 3:** Ablation study of PSMNet.

	EPE	Three-Pixel Error%	Parameters	Running Time
PSMNet infrastructure	1.090	4.346%	5.22 M	500 ms
Increased network compression	1.135	4.864%	3.84 M	452.4 ms
EC-P3D module added	1.187	5.124%	1.72 M	476.2 ms
WReLU added	1.073	3.573%	1.74 M	483.1 ms
Cost volume regularization added	0.770	1.41%	1.74 M	483.1 ms

**Table 4:** Ablation study of AnyNet.

	EPE	Three-Pixel Error%	Parameters	FPS
AnyNet infrastructure	Stage 0 = 5.44, Stage 1 = 4.88, Stage 2 = 4.51	7.25%	34,629	88.1
EC-P3D module added	Stage 0 = 5.79, Stage 1 = 5.12, Stage 2 = 4.74	7.62%	22,683	92.5
WReLU added	Stage 0 = 5.11, Stage 1 = 4.63, Stage 2 = 4.11	6.85%	22,683	91.6

**Table 5:** Experimental results of WReLU in a basic visual network.

Network	Method	Top-1 Accuracy	Top-5 Accuracy	Parameters
SqueezeNet 1.0	Original	57.50%	80.30%	1.25 M
	This Design	64.55%	85.09%	1.25 M
SqueezeNet 1.1	Original	57.10%	80.30%	1.24 M
	This Design	64.08%	84.98%	1.24 M
SqNxt-23	Original	57.80%	80.90%	0.72 M
	This Design	65.15%	86.33%	0.77 M
MobileNetV2	Original	71.88%	90.29%	3.51 M
	This Design	73.80%	91.64%	3.59 M
ShuffleNetV2x05	Original	58.62%	81.14%	1.37 M
	This Design	62.16%	83.45%	1.37 M

## Data Availability

The datasets we used in this study are the KITTI and SceneFlow datasets, and they are openly available at http://www.cvlibs.net/datasets/kitti/ (accessed on 7 August 2022) and https://lmb.informatik.uni-freiburg.de/resources/datasets/SceneFlowDatasets.en.html (accessed on 7 August 2022), respectively.
